# Isorhamnetin Exhibits Hypoglycemic Activity and Targets PI3K/AKT and COX-2 Pathways in Type 1 Diabetes

**DOI:** 10.3390/nu17203201

**Published:** 2025-10-11

**Authors:** Lijia Li, Jia Li, Jie Ren, Jengyuan Yao

**Affiliations:** 1 School of Public Health, Fujian Medical University, Fuzhou 350005, China; lilijia@fjmu.edu.cn (L.L.); 18307940072@fjmu.edu.cn (J.L.); renjie@fjmu.edu.cn (J.R.); 2 Key Laboratory of Functional and Clinical Translational Medicine, Xiamen Medical College, Universities of Fujian Province, Xiamen 361005, China

**Keywords:** isorhamnetin, type 1 diabetes, metabolomics, PI3K/SAKT, eicosanoids, β-cell protection

## Abstract

**Background:** Isorhamnetin (ISO), a dietary O-methylated flavonol, was evaluated for hypoglycemic activity and mechanism in a streptozotocin (STZ) model of type 1 diabetes. **Methods:** We conducted untargeted plasma metabolomics (ESI±), network integration and docking, and measured pancreatic PI3K, phosphorylated AKT, and COX-2; INS-1 β cells challenged with the PI3K inhibitor LY294002 were used to assess viability, intracellular ROS, and PI3K phosphorylation. **Results:** ISO lowered fasting glycemia, increased circulating insulin, improved dyslipidemia by reducing low-density lipoprotein cholesterol (LDL-C), and preserved islet architecture. Untargeted plasma metabolomics (ESI±) indicated broad remodeling with enrichment of arachidonic-, linoleic-, starch/sucrose- and glycerophospholipid pathways. Network integration and docking prioritized targets converging on PI3K/AKT and COX-2/eicosanoid signaling. Consistently, in pancreatic tissue, ISO increased PI3K, phosphorylated AKT, and reduced COX-2. In INS-1 beta cells challenged with the PI3K inhibitor LY294002, ISO improved viability, decreased intracellular ROS, and partially restored PI3K phosphorylation at 4 µM. **Conclusions:** Together, these data indicate that ISO exerts hypoglycemic effects while supporting β-cell integrity through activation of PI3K/AKT and tempering of COX-2–linked lipid-mediator pathways. ISO therefore emerges as a food-derived adjunct candidate for autoimmune diabetes, and the work motivates targeted lipidomics and in vivo pathway interrogation in future studies.

## 1. Introduction

Type 1 diabetes mellitus (T1DM) arises from autoimmune β-cell destruction, producing absolute insulin deficiency and sustained hyperglycemia [1]. Even with modern insulin regimens, durable control is difficult, and complications remain common, which motivates adjunct strategies that stabilize metabolism and protect islet tissue. Against this backdrop, identifying adjunct antihyperglycemic agents remains a priority—not to replace insulin in T1DM, but to complement it, blunt glycemic excursions, and mitigate complications. Major antihyperglycemic drug classes include insulin and, more broadly for diabetes care, metformin, sulfonylureas, thiazolidinediones, α-glucosidase inhibitors, GLP-1 receptor agonists, DPP-4 inhibitors, and SGLT2 inhibitors; however, most non-insulin agents were developed for T2DM and have limited or risk-laden use in T1DM (e.g., ketosis risk with SGLT2 inhibitors).

Isorhamnetin (ISO; 3′-O-methylquercetin) is a dietary flavonol present in Hippophae rhamnoides and Ginkgo biloba and edible plants such as onions and tomatoes, which have been used in traditional diets/remedies with reported antihyperglycemic effects, making ISO a plausible food-derived candidate [2,3]. Beyond its provenance, ISO has been linked to improvements in glucose and lipid handling [2,4]. Mechanistic reports point to several nodes relevant to diabetes: activation of phosphoinositide 3-kinase (PI3K)/AKT [5], engagement of nuclear factor erythroid 2–related factor 2 (Nrf2) with possible AMP-activated protein kinase (AMPK) participation [6], attenuation of cyclooxygenase-2 (COX-2) with implications for arachidonic-acid-derived mediators [7], and promotion of glucose transporter 4 (GLUT4) trafficking in skeletal muscle [4]. These actions support evaluating ISO as an adjunct in insulin-deficient settings.

What remains unclear is whether such effects translate to insulin-deficient diabetes and, specifically, whether ISO can support β-cell integrity via PI3K/AKT while moderating COX-2/eicosanoid signaling in an integrated manner [8,9]. Here, we evaluate ISO in an STZ-induced T1DM model and combine plasma metabolomics with network analysis and β-cell assays to map pathway involvement in the pancreas, focusing on PI3K/AKT and COX-2–linked axes [5,7,10,11]. Given ISO’s occurrence in commonly consumed plants, the findings may inform food-derived strategies for β-cell support [2,3].

We adopted a discovery-to-validation strategy: untargeted plasma metabolomics to nominate pathways, followed by network integration and targeted validation in the pancreas and β-cells under PI3K blockade (LY294002), with weekly fasting blood-glucose trajectories complementing endpoint measures. Our primary objective was to determine whether isorhamnetin (ISO) ameliorates hyperglycemia and preserves islet architecture in an STZ-induced T1DM model; secondarily, we sought to identify ISO-modulated pathways and validate key signals using pharmacological PI3K inhibition as a mechanistic probe. Importantly, this study was prospectively designed as a mechanism-focused investigation of β-cell support, not a head-to-head pharmacological efficacy trial of an antihyperglycemic agent.

## 2. Materials and Methods

### 2.1. Reagents and Chemicals

Isorhamnetin (ISO; ≥98% by HPLC; Shanghai Macklin Biochemical Co., Ltd., Shanghai, China; Cat. No. I811872, commercial standard, not a plant extract) was dissolved in DMSO to prepare a 100 mM stock and was stored at −20 °C, protected from light. For intraperitoneal (i.p.) dosing, working solutions contained 2% DMSO and 20% PEG-400 in PBS; the dose volume was 10 mL/kg. Streptozotocin (STZ, Shanghai Macklin Biochemical Co.) was freshly prepared on ice in 0.1 M sodium-citrate buffer (pH 4.5) and used within 15 min. Dexamethasone (DXM; Solarbio Life Sciences, Beijing, China) was prepared according to the manufacturer’s instructions. LC–MS-grade acetonitrile, methanol, and formic acid were used for metabolomics. Antibodies: P-PI3K (p85 Tyr458/p55 Tyr199; ZenBio, Durham, NC, USA, Cat#310164); PI3K (p85α; Cell Signaling Technology, Danvers, MA, USA, Cat#4292S); COX-2 (Proteintech, Rosemont, IL, USA, Cat#12375-1-AP); BCL-2 (HUABIO, Cambridge, MA, USA, Cat#ET1702-53); PTGER2 (HUABIO, Cat#HA721380); AKT1/2/3 (HUABIO, Cat#ET1609-51); phospho-AKT (Ser473; HUABIO, Cat#ET1607-73); β-actin (HUABIO, Cat#ET1701-80). Secondary antibodies: HRP-conjugated secondary antibodies (HUABIO). Standard reagents (BCA, DCFH-DA, CCK-8, RPMI-1640, trypsin, penicillin–streptomycin) were used as indicated.

### 2.2. Animals and Experimental Design

Specific-pathogen-free male C57BL/6 mice (6–8 weeks, ~20 g) were housed at 22–25 °C, 45–55% humidity, 12 h light/dark, with standard chow and water ad libitum (3–5 per cage, environmental enrichment). Only males were used to reduce estrous-cycle–related variability in this STZ model. All procedures were approved by the Animal Ethics Committee of Xiamen Medical College (No. 20240207015; 7 February 2024) and followed ARRIVE guidelines.

Mice were block-randomized by computer (*n* = 10 per group) into Normal (PBS + PBS), T1DM (STZ 50 mg/kg + PBS), DXM (STZ 50 mg/kg + DXM 10 mg/kg), L-ISO (STZ 50 mg/kg + ISO 20 mg/kg), and H-ISO (STZ 50 mg/kg + ISO 40 mg/kg). Streptozotocin (STZ) is a β-cell–selective nitrosourea transported by GLUT2 that induces DNA alkylation and oxidative stress, causing insulin deficiency and persistent hyperglycemia. We therefore used STZ to generate an insulin-deficient T1DM model in which ISO could be tested, providing a predefined disease background against which ISO’s effects on glycemia and islet morphology could be prospectively evaluated. Dexamethasone (DXM) was included as a mechanistic anti-inflammatory comparator (COX-2/arachidonate repression), not as a glucose-lowering positive control.

After a 1-week acclimation (W−1 to W0), diabetes was induced with STZ i.p. once daily for 7 days during W0–W1 (08:00–10:00). Pharmacological interventions started at W0 and continued once daily for 14 days (W0–W2): ISO (20 or 40 mg/kg) or DXM (10 mg/kg), all given i.p. Following the treatment window, animals were observed drug-free from W2 to W7 to assess durability of effects, and tissues were collected at W7.

Outcome schedule and inclusion criteria. FBG was measured weekly from W0 to W7 after a 12 h fast (baseline, during induction/treatment, and throughout drug-free follow-up). Diabetes induction required FBG ≥ 11.1 mmol/L on two measurements after a 12 h fast. Unless otherwise specified, W0–W7 refers to study week counted from W0. Each group initially included 10 animals (*n* = 10), all monitored for FBG, body weight, and survival. All surviving mice at W7 were analyzed for biochemical indices and organ weights. For histological assessment, three pancreata per group were randomly selected for sectioning and morphometric analysis (≥20 islets per animal). For Western blot validation, pancreatic tissue from three animals per group (*n* = 3) was used.

### 2.3. Biochemical and Histological Analyses

At W7, mice were fasted 12 h and anesthetized with isoflurane (3–5% induction; 1–2% maintenance) on a warming pad. Blood was collected by cardiac puncture; serum was stored at −80 °C. FBG was measured by the glucose-oxidase method with a handheld meter; serum insulin (INS) was quantified by enzyme-linked immunosorbent assay (ELISA). Liver and kidney were weighed to compute organ indices, defined as organ index (%) = organ weight (g)/body weight (g) × 100; the liver index used the whole liver weight, and the renal index used the combined weight of both kidneys. Pancreata were fixed in 4% paraformaldehyde, embedded in paraffin, sectioned at 3 µm and stained with hematoxylin and eosin (H&E). Morphometry used 5 sections per pancreas (150 µm apart), ≥20 islets per animal, and two blinded raters in ImageJ v1.53t.

### 2.4. Untargeted Metabolomics

Plasma (100 µL) was extracted with methanol (1:4, *v*/*v*), vortexed 30 s, ultrasonicated 10 min, incubated at 4 °C for 1 h and centrifuged (15,000 g, 10 min). Supernatants were dried under nitrogen and reconstituted in acetonitrile–water (1:1, *v*/*v*; 100 µL), then analyzed by LC–MS on a Xevo G2-XS QToF with an ACQUITY UPLC BEH C18 column (2.1 mm × 50 mm, 1.8 µm). Mobile phases: 0.01% formic acid in water (A) and acetonitrile (B); gradient 0–1 min 10% B, 1–7.5 min 10–65% B, 7.5–10.5 min 65–100% B, 10.5–12 min 100–10% B; 0.4 mL/min; 1 µL injection. Data were acquired in MSe mode with settings: m/z 50–1200; spray 2.85 kV; source 400 °C; leucine-enkephalin lock mass (m/z 556.2771 for ESI+, and 554.2615 for ESI−). Injections were randomized; blanks were interleaved; and a pooled QC was run every ten injections.

Raw files (MassLynx 4.1) were processed in Progenesis QI (alignment tolerances 10 ppm/0.2 min; adducts +H/−H). Features present in blanks (≥20% of sample intensity) or with quality-control relative standard deviation (QC-RSD) > 30% were removed. Data were normalized by probabilistic quotient normalization (PQN). Statistics (PCA, OPLS-DA, volcano plots) and KEGG pathway enrichment were conducted in MetaboAnalyst 6.0 with Benjamini–Hochberg false discovery rate (FDR) control (q < 0.05). Model validity was checked by 7-fold cross-validation, CV-ANOVA (*p* < 0.01) and 1000 permutations.

### 2.5. Network Pharmacology and Molecular Docking

Putative ISO targets were retrieved from SwissTargetPrediction (probability ≥ 0.1), and PharmMapper (max conformers = 300; pharmacophore models with pKd ≥ 6.0); STRING v11.5 PPI networks were built at confidence ≥ 0.7. T1DM-associated genes were compiled from GeneCards (relevance ≥ 1), OMIM and TTD. Queries were performed in May 2025; target names were de-duplicated and standardized via UniProt. Overlapping targets were submitted to STRING v11.5 (Homo sapiens; confidence ≥ 0.7) to build the protein–protein interaction (PPI) network and analyzed in Cytoscape 3.9.0 (CentiScape). Gene Ontology (GO), Kyoto Encyclopedia of Genes and Genomes (KEGG), and Reactome enrichment were performed in DAVID and Reactome, with Benjamini–Hochberg FDR < 0.05 considered significant.

For docking, ISO was prepared in its protonation state at pH 7.4. Representative human structures (resolution ≤ 3.0 Å) for hub proteins (e.g., AKT1, PTGS2, PPARG, EGFR, ESR1, SRC) were obtained from the RCSB Protein Data Bank (PDB). The CB-DOCK2 server predicted binding cavities.

### 2.6. Western Blotting

Pancreatic tissue or INS-1 cells were lysed in radioimmunoprecipitation assay (RIPA) with phenylmethylsulfonyl fluoride (PMSF) and phosphatase inhibitors. Protein was quantified by BCA. Equal amounts (10–20 µg) were separated by SDS–PAGE (10–12%) and transferred to polyvinylidene difluoride (PVDF). Membranes were blocked (5% milk/TBST, 1 h, RT), incubated with primary antibodies (1:1000; β-actin 1:5000) overnight at 4 °C, then with HRP-conjugated secondary antibodies (1:5000) for 1 h. Chemiluminescence was captured on a ChemStudio 515 imaging system (Analytik Jena, Germany) within the linear range; densitometry (ImageJ) was normalized to β-actin, which did not differ across groups. Each “n” denotes a biological replicate; for pancreatic Western blots, n = 3 animals per group.

### 2.7. Cell-Based Assays

INS-1 β-cells (RRID: CVCL_0351; authenticated, mycoplasma-free) were maintained in RPMI-1640 with 10% FBS, 1% penicillin–streptomycin and 50 µM β-mercaptoethanol at 37 °C, 5% CO_2_ in a humidified incubator. Cells (2 × 10^4^ per well) were seeded in 96-well plates 24 h before treatment. LY294002 (5, 10, 15 µM) was applied for 24 h with or without ISO (1, 2, 4 µM). For clarity, the experimental groups were: Vehicle (0.1% DMSO); ISO 1 µM; ISO 2 µM; ISO 4 µM; LY294002 5 µM (LY5); LY294002 10 µM (LY10); LY294002 15 µM (LY15); LY5 + ISO1; LY5 + ISO2; LY5 + ISO4; LY10 + ISO1; LY10 + ISO2; LY10 + ISO4; LY15 + ISO1; LY15 + ISO2; LY15 + ISO4. All conditions were matched for final DMSO (≤0.1%). Unless otherwise indicated in figure legends, panels illustrating PI3K blockade use LY294002 at 10 µM (LY10). Viability was measured by Cell Counting Kit-8 (CCK-8) at 450 nm. Intracellular ROS was assessed with 10 µM 2′,7′-dichlorodihydrofluorescein diacetate (DCFH-DA) (30 min, 37 °C, dark) with excitation/emission at 488/525 nm, and ROS signals were normalized to per-well viability. Each condition included ≥3 biological replicates (independent passages), each with 3 technical wells.

### 2.8. Statistical Analysis

Data are reported as mean ± SEM unless otherwise stated. Comparisons between two or more groups used parametric or non-parametric tests as appropriate. Normality and homoscedasticity were assessed using Shapiro–Wilk and Levene tests. Analyses then followed assumption-based branches: if data were normal with equal variances, Student’s *t*-test or one-way ANOVA with Tukey’s post hoc was used; if data were non-normal, Mann–Whitney U or Kruskal–Wallis with Dunn’s post hoc was used; if variances were unequal, Welch’s *t*-test or Welch’s ANOVA with Games–Howell was used. Exact *p*-values are reported where space permits. For multiple endpoints, Holm–Šidák adjustment was applied; for metabolomics, FDR was controlled at q < 0.05. Orthogonal partial least squares–discriminant analysis (OPLS-DA) models were validated by 7-fold cross-validation, cross-validation analysis of variance (CV-ANOVA) (*p* < 0.01) and 1000 permutations.

### 2.9. Prespecified Endpoints and Dose Rationale

Primary endpoint: fasting blood glucose (FBG). Secondary endpoints: serum insulin, LDL-C/HDL-C, liver/kidney indices, and relative islet area. Mechanistic endpoints: plasma metabolomics (arachidonic-acid, linoleic-acid, glycerophospholipid and carbohydrate pathways), pancreatic PI3K/AKT and COX-2 proteins, and β-cell assays under PI3K inhibition (LY294002) evaluating viability and intracellular ROS. Dose and route rationale: ISO was administered i.p. at 20 or 40 mg/kg for 14 days to minimize exposure variability from oral absorption and to remain within commonly used in vivo ranges aligned with PI3K/AKT and eicosanoid-linked mechanisms. The dosing vehicle contained 2% DMSO and 20% PEG-400 in PBS.

## 3. Results

### 3.1. ISO Improves Glycemic Control, Lipid Profile, and Islet Integrity in T1DM Mice

**Figure 1:**
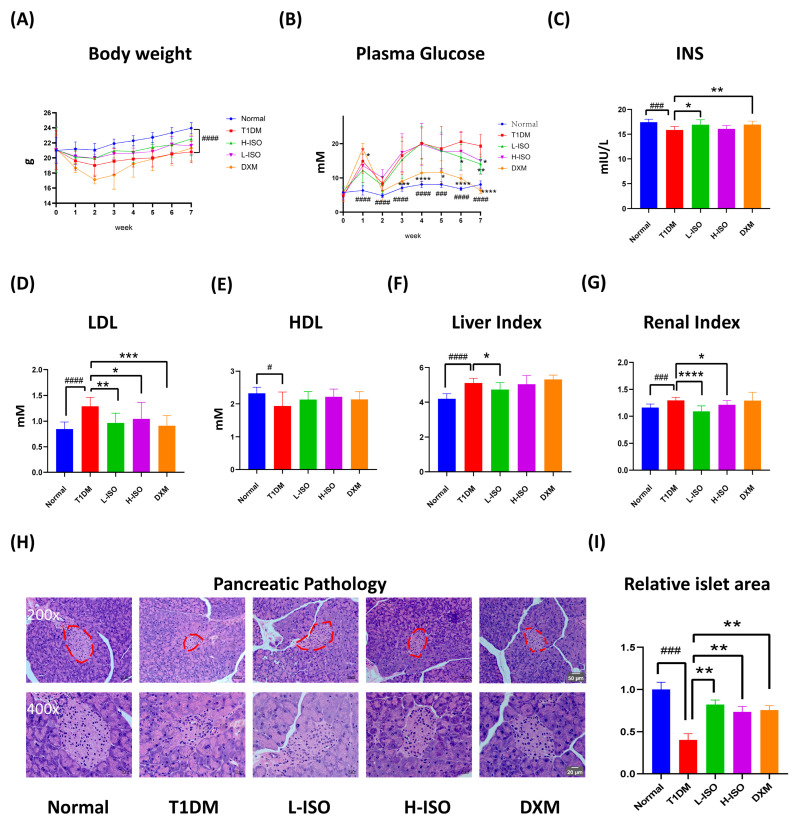
ISO improves glycemia, lipid profile, and islet morphology in STZ-induced T1DM mice. (**A**) Weekly body weight and (**B**) weekly fasting blood-glucose (FBG) trajectories from W0 to W7; the treatment window (W0–W2) is shaded. Hashes (#) denote comparisons versus Normal, asterisks (*) denote comparisons versus T1DM; symbols are plotted on the colored line of the contrasted group. (**C**) Plasma insulin at the W7 endpoint. (**D**,**E**) Serum LDL-C and HDL-C. (**F**,**G**) Liver and kidney indices. (**H**) Representative pancreatic H&E sections (200× and 400×). (**I**) Relative islet area at W7. Data are mean ± SEM (n = 10 per group). * *p* < 0.05, ** *p* < 0.01, *** *p* < 0.001, **** *p* < 0.0001 vs. T1DM; # *p* < 0.05, ### *p* < 0.001, #### *p* < 0.0001 vs. Normal.

In STZ-induced diabetic mice, marked metabolic disturbances were observed versus Normal, including significant weight loss (*p* < 0.0001) and elevated fasting blood glucose (FBG; *p* < 0.0001). Weekly FBG trajectories (Figure 1B) from W0 to W7 showed sustained hyperglycemia in T1DM; ISO showed a delayed but significant effect, first evident at W6 in L-ISO and W7 in H-ISO, with L-ISO providing the more consistent control. At the W7 endpoint, ISO significantly lowered FBG (L-ISO *p* < 0.01; H-ISO *p* < 0.05) and increased plasma insulin in L-ISO (*p* < 0.05; Figure 1B,C). Lipid profiling confirmed elevated LDL-C and reduced HDL-C in T1DM mice; ISO reduced LDL-C (L-ISO *p* < 0.01; H-ISO *p* < 0.05), while HDL-C showed a non-significant upward trend (Figure 1D,E). Organ indices were altered: the T1DM group exhibited an increased liver index versus Normal (*p* < 0.0001) and an increased renal index (*p* < 0.001); ISO mitigated these changes, with L-ISO reducing both indices and H-ISO improving the renal index (Figure 1F,G). Histology (Figure 1H,I) showed that T1DM islets were shrunken with disorganized cords, whereas ISO preserved islet size and contour and improved cellularity. Dexamethasone (DXM) was included as a mechanistic anti-inflammatory comparator (COX-2/arachidonate repression), not as an antihyperglycemic positive control.

### 3.2. ISO Modulates Metabolic Profiles and Key Pathways in T1DM

**Figure 2:**
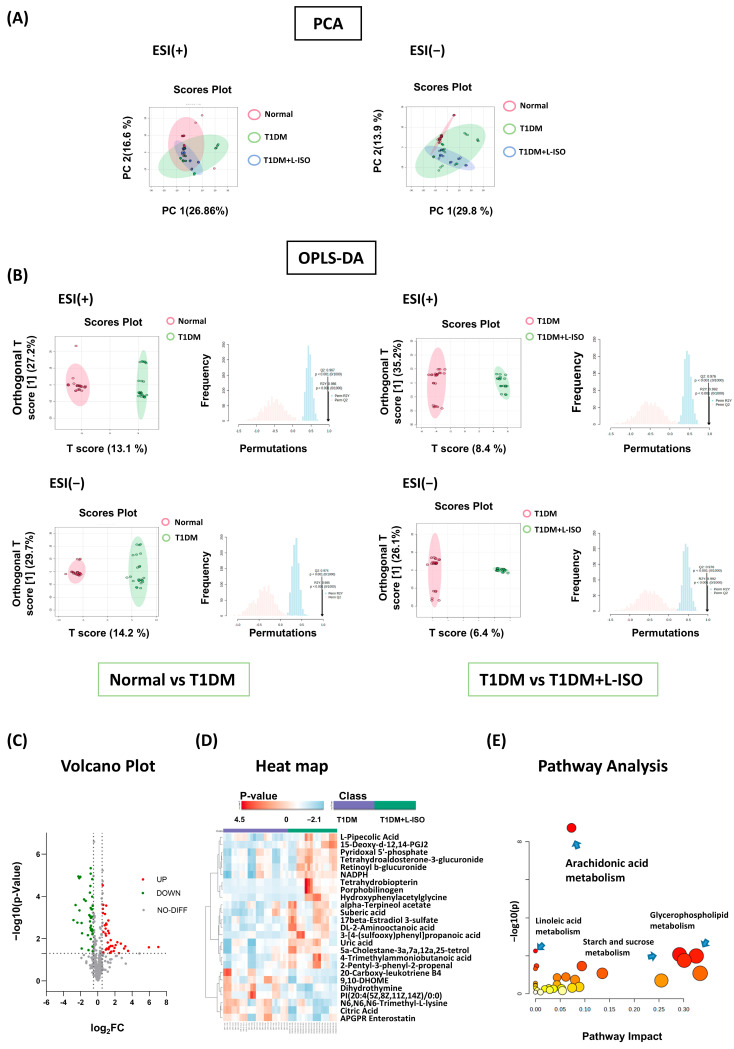
ISO reprograms the plasma metabolome of diabetic mice. (**A**) PCA score plots (ESI^+^/ESI^−^) for Normal (pink), T1DM (green), and T1DM + L-ISO (blue). (**B**) OPLS-DA score plots for pairwise models: Normal vs. T1DM (left) and T1DM vs. T1DM + L-ISO (right); adjacent histograms show permutation-test Q^2^ distributions (1000 permutations), with the downward arrow marking the observed (non-permuted) Q^2^; of the original model. (**C**) Volcano plot for T1DM vs. T1DM + L-ISO (*p* < 0.05, |fold change| > 1.5). (**D**) Heatmap of the top-25 VIP metabolites (same comparison). (**E**) KEGG pathway enrichment of differential metabolites; bubble size indicates metabolite count and color denotes −log_10_(*p*). Analyses were performed in MetaboAnalyst 6.0.

Untargeted metabolomics detected 4260 and 3908 ion features in ESI^+^ and ESI^−^ modes, respectively (Tables S1 and S2). Annotation with the Human Metabolome Database (HMDB) yielded putative identities for 255 metabolites (ESI^+^) and 288 (ESI^−^). Principal-component analysis (PCA) showed groupwise shifts with partial separation among Normal, T1DM, and T1DM+L-ISO, indicating systemic metabolic remodeling under disease and treatment (Figure 2A). Supervised OPLS-DA further achieved clear discrimination in both ion modes for Normal vs. T1DM and for T1DM vs. T1DM+L-ISO, with permutation testing (1000 runs) supporting model robustness (R^2^Y and Q^2^ both high, no overfitting; Figure 2B).

Comparing T1DM with T1DM+L-ISO, volcano analysis identified 79 differential metabolites (*p* < 0.05, fold change > 1.5), including 40 down- and 39 up-regulated features after ISO treatment (Figure 2C). The top-25 VIP (variable importance in projection) heatmap confirmed broad reversal of T1DM-disrupted patterns (Figure 2D). KEGG pathway enrichment (FDR q < 0.05) highlighted arachidonic-acid, linoleic-acid, starch/sucrose and glycerophospholipid metabolism as major ISO-responsive routes (Figure 2E), pathways closely linked to inflammatory signaling and energy homeostasis. Putative biomarkers with AUC > 0.8 are summarized in Table 1 (e.g., porphobilinogen, 20-carboxy-LTB4, 6-keto-PGF1α, 15-deoxy-Δ12,14-PGJ2).

### 3.3. Network Pharmacology and Molecular Docking Highlight PI3K/AKT as a Central ISO Target

**Figure 3:**
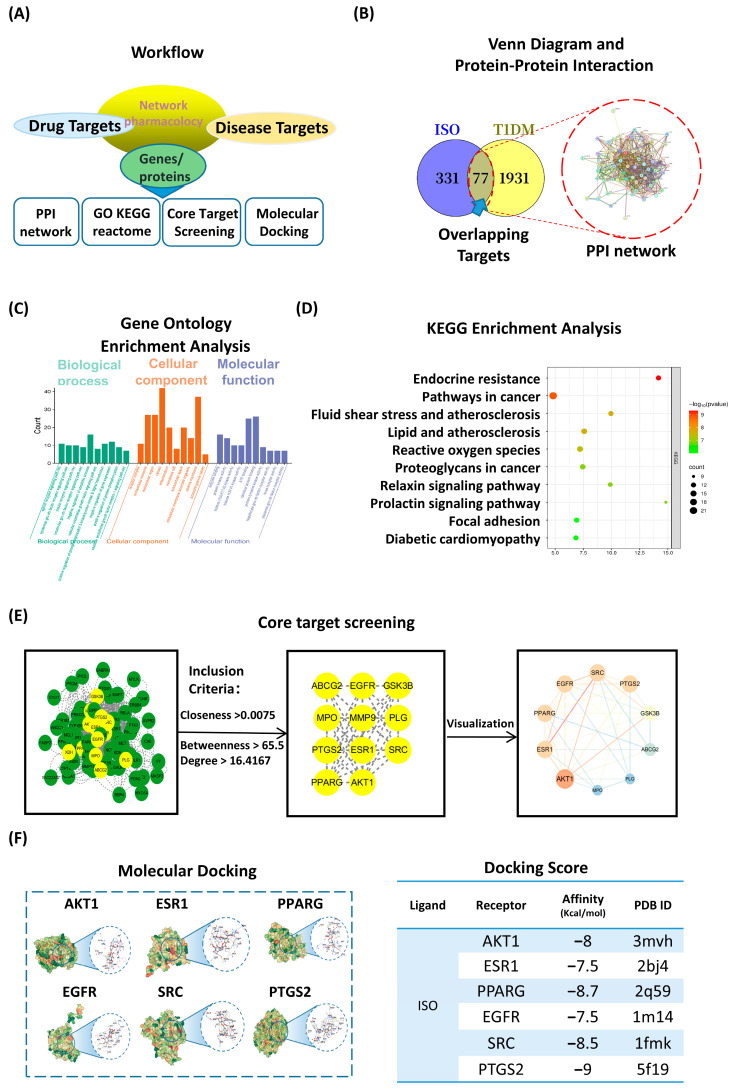
ISO targets the PI3K/AKT axis according to network-pharmacology and molecular-docking analyses. (**A**) Workflow: ISO and T1DM targets were collected and integrated via PPI construction, enrichment analysis, topological screening, and docking. (**B**) Venn diagram showing 77 overlapping targets; inset, STRING PPI network (72 nodes, 591 edges). (**C**) GO enrichment (top 10 terms for BP/CC/MF). (**D**) KEGG enrichment bubble plot; bubble size indicates gene count and color denotes −log_10_(*p*). (**E**) Core-target screening based on topology (closeness > 0.0075; betweenness > 65.5; degree > 16.4) with the interaction subnetwork. (**F**) Docking to six hubs (AKT1, ESR1, PPARG, EGFR, SRC, PTGS2); representative poses (left) and binding affinities (kcal/mol) with PDB IDs (right). Corresponding 2D interaction diagrams are provided in Supplementary Figure S2.

Network pharmacology workflow integrated compound and disease targets (Figure 3A). We collected 331 putative ISO targets and 1931 T1DM-associated genes; their intersection yielded 77 overlapping targets (Figure 3B). The STRING PPI network (72 nodes, 591 edges) indicated substantial interconnectivity. Gene Ontology (GO) enrichment implicated insulin-receptor signaling and positive regulation of PI3K/AKT (BP), receptor complexes/extracellular vesicles (CC), and kinase/ATP binding (MF) (Figure 3C). KEGG pathway enrichment highlighted endocrine resistance, fluid shear stress and atherosclerosis, chemical carcinogenesis—reactive oxygen species, proteoglycans in cancer, relaxin/prolactin signaling, focal adhesion, and diabetic cardiomyopathy—pathways that converge on PI3K/AKT nodes (Figure 3D).

Topological screening (degree > 16.4, betweenness > 65.5, closeness > 0.0075) identified 12 hubs, including AKT1, PTGS2, PPARG, EGFR, ESR1 and SRC (Figure 3E). Docking supported favorable interactions with these hubs (−7.5 to −9.0 kcal/mol), consistent with stable binding poses (Figure 3F). These network-pharmacology and docking results are hypothesis-generating and guided targeted validation (Table 2).

### 3.4. ISO Coordinates Gene–Metabolite Networks to Regulate Inflammation and Cell Survival

**Figure 4:**
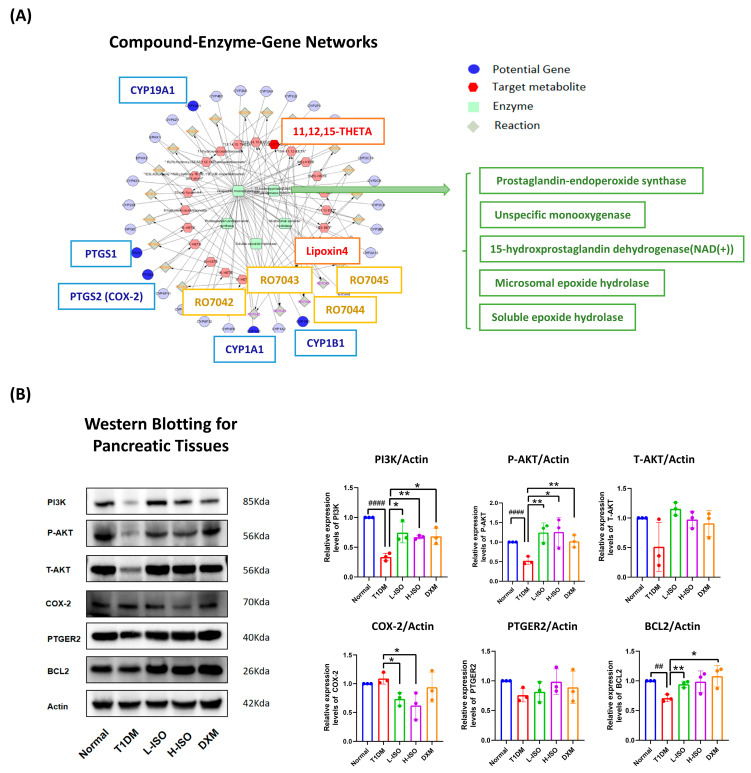
ISO integrates metabolite and gene networks to modulate inflammatory and survival signaling. (**A**) Simplified compound–enzyme–gene subnetwork focused on the arachidonic-acid/eicosanoid axis, highlighting key ISO-linked hubs and metabolites (e.g., PTGS2/COX-2, CYP1A1/1B1, CYP19A1, 11,12,15-THETA, lipoxin A4). Labels are enlarged for legibility; nodes are color-coded by four concentric layers. The full network and parameter settings are provided in Supplementary Figure S1 (vector file); the underlying data are available as Supplementary Tables S3 and S4 (node and edge tables, CSV). (**B**) Western blot validation of PI3K, phospho-AKT (P-AKT), total AKT (T-AKT), BCL-2, COX-2, and PTGER2 in pancreatic tissue. Bar graphs show band intensities normalized to actin (mean ± SEM, n = 3). Significance codes: * *p* < 0.05, ** *p* < 0.01 (vs. T1DM); ## *p* < 0.01, #### *p* < 0.0001 (vs. Normal). Colored dots over each bar denote individual biological replicates (n = 3); dot colors match the corresponding bar colors.

To integrate metabolomic and network-pharmacologic insights, we constructed a compound–reaction–enzyme–gene network (Figure 4A; Supplementary Figure S1; Tables S3 and S4), linking ISO-responsive plasma metabolites to candidate targets via enzymes and reactions. A representative subnetwork enriched for arachidonic-acid–related nodes highlighted connections between eicosanoid metabolism and cell-survival signaling.

In pancreatic tissue, T1DM significantly reduced PI3K, P-AKT, and BCL-2 (*p* < 0.0001, *p* < 0.0001, *p* < 0.01 vs. Normal). L-ISO increased PI3K (*p* < 0.05) and P-AKT (*p* < 0.01) and lowered COX-2 (*p* < 0.05) relative to T1DM. H-ISO similarly elevated PI3K (*p* < 0.01) and P-AKT (*p* < 0.05) and reduced COX-2 (*p* < 0.05). T-AKT and PTGER2 exhibited upward but non-significant trends. Dexamethasone (DXM) produced a comparable profile, increasing PI3K, P-AKT, and BCL-2 (*p* < 0.05, *p* < 0.01, *p* < 0.05). These data align with network-level predictions and support ISO-mediated activation of PI3K/AKT alongside suppression of COX-2.

### 3.5. ISO Protects INS-1 β Cells from LY294002-Induced Dysfunction by Enhancing Viability, Reducing ROS, and Supporting PI3K/AKT Signaling

**Figure 5:**
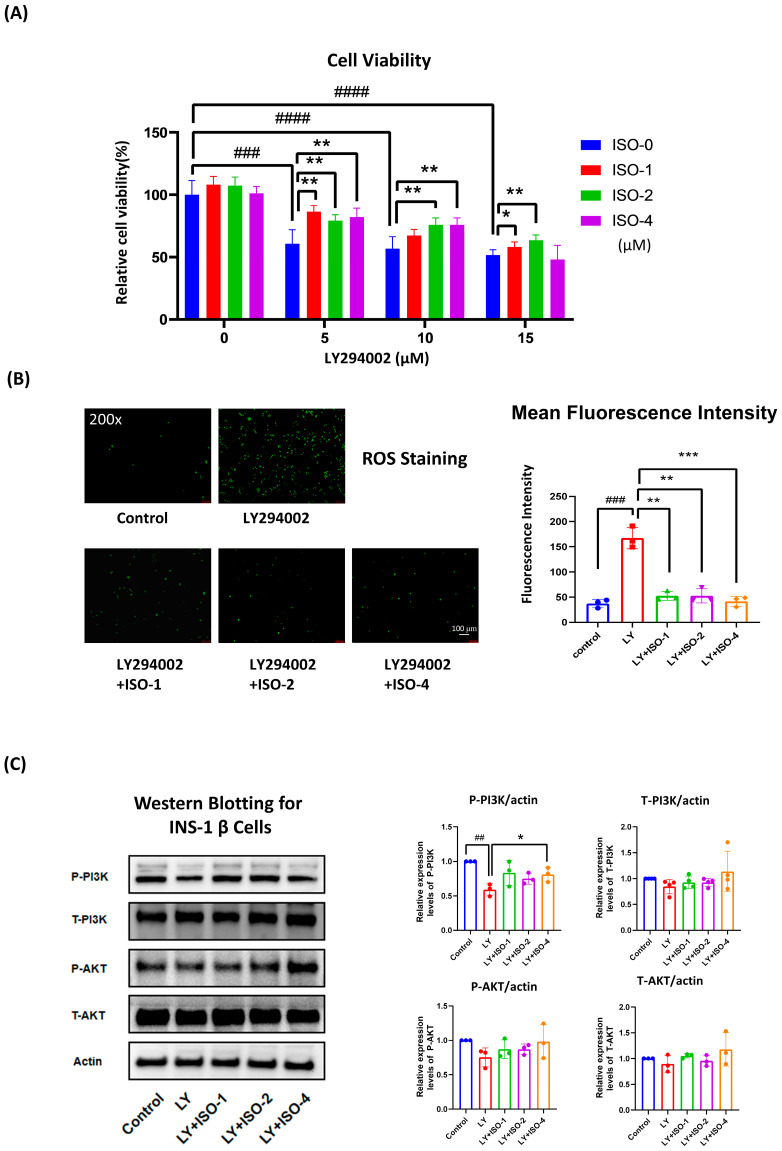
ISO protects INS-1 β-cells from PI3K inhibition–induced damage. (**A**) Cell viability (CCK-8) after 24 h co-treatment with LY294002 (0, 5, 10, 15 µM) and ISO (0, 1, 2, 4 µM). (**B**) Intracellular ROS visualized by DCFH-DA (representative micrographs) and quantified. (**C**) Western blot of PI3K/AKT signaling: phospho-PI3K (P-PI3K), total PI3K (T-PI3K), phospho-AKT (P-AKT), and total AKT (T-AKT), with β-actin as the loading control. Bars show densitometry normalized to actin (mean ± SEM, n = 3). * *p* < 0.05, ** *p* < 0.01, *** *p* < 0.001 vs. LY-only; ## *p* < 0.01, ### *p* < 0.001, #### *p* < 0.0001 vs. untreated Control. Colored dots overlaid on bars denote individual biological replicates (n = 3).

To assess the protective effects of ISO in pancreatic β cells, we established an injury model using the PI3K inhibitor LY294002. As shown in Figure 5A, LY294002 significantly reduced INS-1 viability in a dose-dependent manner (5, 10, and 15 µM; *p* < 0.001, *p* < 0.0001, and *p* < 0.0001, respectively). Co-treatment with ISO (1, 2, or 4 µM) improved viability in a concentration- and stress-dependent fashion. Under 5 µM LY, all ISO doses significantly restored viability (*p* < 0.01, *p* < 0.01, *p* < 0.01). At 10 µM LY, ISO at 2 and 4 µM remained effective (*p* < 0.01), while at 15 µM LY, 1 and 2 µM ISO were protective (*p* < 0.05 and *p* < 0.01, respectively).

ROS imaging (Figure 5B) showed that LY294002 markedly increased intracellular oxidative stress (*p* < 0.001), which was significantly reduced by ISO at all concentrations (*p* < 0.01), indicating an antioxidant effect. Western blotting (Figure 5C) showed suppression of P-PI3K by LY294002 (*p* < 0.01); ISO at 4 µM significantly reversed this reduction (*p* < 0.05), with lower doses showing mild recovery. Total PI3K, P-AKT, and T-AKT displayed slight, non-significant increases with ISO. Collectively, ISO counteracts PI3K inhibition–induced dysfunction by enhancing viability, attenuating ROS, and supporting PI3K/AKT pathway activity.

## 4. Discussion

Isorhamnetin (ISO) alleviates hyperglycemia in STZ-induced T1DM, lowers LDL-C, increases plasma insulin (L-ISO), and preserves islet morphology (significant in L-ISO; Figure 1). This dose–response pattern is compatible with a biphasic (hormetic) window. At lower exposure, ISO preferentially supports PI3K/AKT signaling and tempers COX-2–eicosanoid activity, whereas higher exposure may reach pathway saturation and introduce off-target/redox liabilities, blunting net benefit. Non-linear exposure after i.p. dosing of a poorly water-soluble flavonol could further contribute (e.g., variable absorption/distribution despite matched vehicle). Consistent with this, L-ISO achieved steadier glycemic control in vivo and restored P-PI3K at 4 µM in INS-1 cells, while H-ISO did not confer additional advantages. Future work will include PK/PD profiling and a finer dose grid (10–60 mg/kg) to delineate the therapeutic window and separate pharmacodynamic from exposure-driven effects. Untargeted metabolomics indicates broad remodeling with enrichment of arachidonic-acid, linoleic-acid, starch/sucrose and glycerophospholipid pathways (Figure 2). Network pharmacology highlights AKT1, PTGS2, PPARG, EGFR, ESR1 and SRC as hubs, with docking supporting favorable ISO binding (Figure 3). Mechanistically, ISO supports PI3K/AKT signaling in pancreatic tissue (increased P-AKT) and reduces COX-2 (Figure 4), and in INS-1 cells counters LY294002-induced dysfunction by improving viability, lowering ROS and restoring P-PI3K at 4 µM (Figure 5). Contextualization with recent literature: studies have shown that ISO and related flavonols can modulate glucose homeostasis, β-cell stress responses, and inflammatory eicosanoid signaling in metabolic and diabetic models; our findings extend these observations to an insulin-deficient STZ model and connect the phenotypes to a PI3K/AKT–COX-2/eicosanoid framework [5,8,9]. Network-pharmacology and docking findings are hypothesis-generating and were used to guide targeted validation rather than to claim direct target engagement in vivo.

The PI3K/AKT pathway is a canonical survival axis in β-cells. Genetic activation of Akt1 enlarges β-cells, enhances proliferation, and limits apoptosis, thereby expanding β-cell mass in independent transgenic models [12,13]. Complementary loss-of-function evidence shows that deleting class-IA PI3K subunits impairs insulin secretion and β-cell performance, whereas re-expression of constitutively active Akt normalizes these defects—highlighting that PI3K signals converge on AKT to maintain β-cell fitness [14]. At the cellular level, glucose promotes β-cell survival via PI3K/AKT; pharmacologic PI3K inhibition abolishes glucose-induced Akt phosphorylation and cytoprotection, while constitutively active Akt rescues survival under stress [15]. In this context, our data—ISO elevating pancreatic P-AKT in vivo and partially restoring P-PI3K and viability in LY294002-challenged INS-1 cells—support a model in which ISO preserves β-cell integrity primarily by sustaining PI3K/AKT signaling. These observations are consistent with recent reports underscoring PI3K/AKT as a central survival node in β-cells and a tractable target of flavonols in vivo [16,17].

Our metabolomics enrichment in arachidonic-acid and glycerophospholipid pathways, together with reduced pancreatic COX-2 and a trend toward higher PTGER2, suggests a reset of eicosanoid tone in ISO-treated mice (Figure 2 and Figure 4). In pancreatic islets, COX-2-derived PGE_2_ signals through receptor-specific routes with divergent consequences: EP3 (PTGER3) is induced in diabetic islets and suppresses cAMP/GSIS, thereby impairing β-cell function [18], whereas EP4 engagement promotes β-cell survival; accordingly, EP3 blockade augments survival and proliferation in mouse and human islets ex vivo [19]. Upstream lipid-liberating enzymes couple this axis to secretion—Group X secretory phospholipase A_2_ increases PGE_2_ and depresses GSIS via a COX-2/EP3-dependent mechanism, reversible by a COX-2 inhibitor or EP3 antagonist [20]. In human islets, PGE_2_-synthetic enzymes are elevated and positively correlate with markers of β-cell function/mass in nondiabetic obesity, indicating pathway activity in human tissue and context-dependent effects dictated by receptor balance [21]. Together with prior evidence that PTGS2–PTGER2/4 signaling can protect against STZ injury in vivo [22], these observations support a model in which ISO attenuates COX-2 and may shift signaling toward EP2/EP4, relieve EP3-Gi–cAMP inhibition while favoring survival pathways, aligning with improved islet architecture and β-cell signaling. Recent studies likewise link the COX-2/EP-receptor balance to β-cell function and survival, supporting the idea that modulation of EP3–Gi while favoring EP2/EP4 contributes to improved islet outcomes [21,23,24,25].

β-cells are highly vulnerable to reactive oxygen/nitrogen species, and here ISO lowered intracellular ROS across doses in INS-1 cells (Figure 5B). Isorhamnetin is known to activate the Keap1–Nrf2 antioxidant program and induce canonical targets (e.g., HO-1, NQO1) [6]. In β-cells specifically, genetic gain- and loss-of-function studies show that Nrf2 restrains oxidative damage, preserves insulin secretion, and expands functional β-cell mass in vivo and in human islet xenografts [26,27]. Mechanistically, PI3K/AKT and Nrf2 likely cooperate: AKT inhibits GSK-3β, a kinase that promotes Fyn-dependent nuclear export/degradation of Nrf2; thus, AKT activation stabilizes nuclear Nrf2 and sustains antioxidant transcription [28,29]. Taken together, ISO’s simultaneous enhancement of PI3K/AKT signaling and suppression of ROS supports a framework in which Nrf2-mediated cytoprotection amplifies the β-cell survival benefits conferred by PI3K/AKT, consistent with the improved glycemia and islet morphology observed in vivo. This integrated PI3K/AKT–Nrf2–eicosanoid view provides a coherent mechanistic basis for ISO’s β-cell protection and aligns with recent preclinical evidence [27,30].

This study has several limitations. First, although ISO increased pancreatic P-AKT in vivo and restored P-PI3K under LY294002 in INS-1 cells, causal attribution to the PI3K/AKT axis in the animal model remains inferential; in vivo perturbation (e.g., pharmacological or genetic) would strengthen mechanistic certainty [10,12,13,14,15]. Second, the eicosanoid arm was inferred from untargeted metabolomics plus COX-2 down-regulation; targeted lipidomics of prostaglandins/leukotrienes (e.g., PGE_2_, 15-deoxy-Δ^12^,^14^-PGJ_2_, 6-keto-PGF_1_α) and receptor-level modulation would directly anchor the COX-2–PTGER axis to outcomes [7,11,18,19,20,21,22]. Third, dosing used a short, 14-day i.p. regimen; pharmacokinetics, tissue exposure, and oral bioavailability/formulation remain to be defined, despite the dietary occurrence of ISO [2,3,5]. Fourth, our apoptosis panel was limited: beyond a small BCL-2 change, we did not measure other BCL-2 family regulators (e.g., MCL-1, BAX, BCL-XL) or cleaved caspase-3; future work will include these targets in WB/IHC to determine whether ISO shifts the apoptotic balance. Fifth, we did not quantify peri-islet immune infiltrates or profile T-cell subsets; future work will include IHC/flow cytometry of CD3, CD4 (Th), CD8 (Tc), Foxp3 (Treg), and TCRγδ T cells, together with myeloid markers (F4/80, CD68), to map ISO-related immune changes. Additionally, dynamic glycemic readouts (OGTT/IPGTT) and an in vivo insulin-treated pharmacological positive control were not included; an independent validation cohort will incorporate these elements alongside exposure profiling and dose-window mapping to strengthen translational inference [31,32,33,34].

In summary, our data position ISO as a potential adjunct to insulin therapy by attenuating hyperglycemia while supporting β-cell integrity. Mechanistically, the discovery-to-validation sequence converges on PI3K/AKT activation together with COX-2/eicosanoid tempering, a combination linked to improved islet survival and inflammatory tone. We did not perform dynamic glycemia testing (OGTT/IPGTT) in the present study; an independent validation cohort will include these indices together with an insulin-treated benchmark to compare translational efficacy.

## 5. Conclusions

Isorhamnetin ameliorates hyperglycemia and dyslipidemia, improves plasma insulin, and preserves islet architecture in STZ-induced T1DM, while being associated with metabolite remodeling enriched for arachidonic- and glycerophospholipid-linked pathways. Multi-layer analyses support a dual-axis mechanism in the pancreas: support of PI3K/AKT signaling and attenuation of COX-2-dependent lipid-mediator signaling complemented by antioxidant support, consistent with Nrf2 engagement. Together, these findings position ISO as a food-derived adjunct of β-cell resilience and inflammatory tone, warranting targeted lipidomics, in vivo pathway testing, and the inclusion of dynamic glycemic indices with an insulin benchmark in a validation cohort, as well as oral-formulation studies to advance translational potential.

## Figures and Tables

**Table 1:** Key metabolites altered by ISO treatment in T1DM mice (T1DM+L-ISO vs. T1DM comparison; ESI^+^/ESI^−^).

Description	ESI Mode	*m*/*z*	Retention Time (min)	HMDB ID	KEGG ID	PubChem ID	AUC	*p* Value	Fold Change (log_2_)
Porphobilinogen	positive	227.1048	1.28	HMDB0000245	C00931	1021	0.97	0.00026	−2.5903
20-Carboxy-leukotriene B4	negative	365.1983	0.92	HMDB0006059	C05950	5280877	0.91	0.00138	1.2271
Cinnamic acid	positive	149.0590	0.77	HMDB0000567	C10438	5372954	0.91	0.00000	−0.4465
Sphinganine	positive	302.3061	1.80	HMDB0000269	C00836	91486	0.90	0.00007	0.5605
6-keto-prostaglandin F1α	positive	371.2367	3.47	HMDB0002886	C05961	5280888	0.89	0.00001	−0.8282
Tetrahydrobiopterin	positive	242.1202	1.45	HMDB0000027	C00272	44257	0.88	0.00133	−2.8345
3-(Methylthio)-1-propanol	positive	107.0510	1.08	HMDB0031716	C08249	10448	0.86	0.00023	0.4962
Glycerol 3-phosphate	negative	171.0070	0.80	HMDB0000126	C00093	439162	0.85	0.00243	−0.7937
Citric acid	negative	191.0201	0.75	HMDB0000094	C00158	311	0.85	0.00003	0.5885
Pyridoxal 5’-phosphate	positive	248.0350	1.13	HMDB0001491	C00018	1051	0.85	0.00007	−0.9147
15-Deoxy-d-12,14-PGJ2	positive	317.2095	3.24	HMDB0005079	C14717	5311211	0.84	0.00001	−2.1549
PE(20:4(5Z,8Z,11Z,14Z)/18:0)	negative	766.5385	4.30	HMDB0009387	C00350	52924644	0.84	0.00154	−0.3945
Uric acid	negative	167.0216	0.73	HMDB0000289	C00366	1175	0.83	0.00010	−0.6480
N6,N6,N6-Trimethyl-L-lysine	positive	189.1610	1.00	HMDB0001325	C03793	440120	0.81	0.00027	0.9815
Suberic acid	negative	173.0829	0.22	HMDB0000893	C08278	10457	0.81	0.00041	−0.7202
Retinoyl β-glucuronide	positive	477.2401	0.84	HMDB0003141	C11061	5281877	0.81	0.00035	−0.8292
12,13-DiHOME	negative	313.2374	1.25	HMDB0004705	C14829	25320870	0.80	0.00025	0.64733

Note. AUC, area under the ROC curve; *m*/*z*, mass-to-charge ratio; HMDB, Human Metabolome Database; KEGG, Kyoto Encyclopedia of Genes and Genomes; PubChem ID, compound identifier in PubChem. “Positive/negative” denote electrospray-ionization mode. Fold change is log_2_(ISO/T1DM); negative values indicate lower abundance in ISO relative to T1DM (downregulation by ISO).

**Table 2:** Top 10 Reactome pathways enriched by overlapping ISO–T1DM targets.

Pathway Name	*p* Value	FDR	Pathway Identifier
Interleukin-4 and Interleukin-13 signaling	8.22 × 10^−14^	7.88 × 10^−11^	R-HSA-6785807
Negative regulation of the PI3K/AKT network	7.45 × 10^−13^	3.57 × 10^−10^	R-HSA-199418
PI3K/AKT Signaling in Cancer	3.25 × 10^−12^	1.04 × 10^−9^	R-HSA-2219528
PI5P, PP2A and IER3 Regulate PI3K/AKT Signaling	5.30 × 10^−12^	1.27 × 10^−9^	R-HSA-6811558
Extra-nuclear estrogen signaling	6.66 × 10^−12^	1.27 × 10^−9^	R-HSA-9009391
Signaling by Receptor Tyrosine Kinases	2.10 × 10^−11^	3.33 × 10^−9^	R-HSA-9006934
ESR-mediated signaling	2.14 × 10^−10^	2.94 × 10^−8^	R-HSA-8939211
PIP3 activates AKT signaling	4.85 × 10^−10^	5.53 × 10^−8^	R-HSA-1257604
Intracellular signaling by second messengers	5.21 × 10^−10^	5.53 × 10^−8^	R-HSA-9006925
Constitutive Signaling by Aberrant PI3K in Cancer	7.75 × 10^−10^	7.36 × 10^−8^	R-HSA-2219530

Note. Pathways are ranked by ascending *p* value; FDR denotes Benjamini–Hochberg-adjusted values; “Pathway identifier” lists the Reactome stable IDs.

## Data Availability

The original contributions presented in this study are included in the article/Supplementary Materials. Further inquiries can be directed to the corresponding author.

## Supplementary Materials

The following supporting information can be downloaded at: https://www.mdpi.com/article/10.3390/nu17203201/s1, Figure S1: Full compound–enzyme–gene network; Figure S2: Docking interaction details; Table S1: LC–MS/MS compounds (positive mode); Table S2: LC–MS/MS compounds (negative mode); Table S3: Node table for the compound–reaction–enzyme–gene network; Table S4: Edge table for the compound–reaction–enzyme–gene network.

## Abbreviations

The following abbreviations are used in this manuscript:

AKT	protein kinase B
AMPK	AMP-activated protein kinase
CCK-8	Cell Counting Kit-8
COX-2	cyclooxygenase-2
CV-ANOVA	cross-validation analysis of variance
DCFH-DA	2′,7′-dichlorodihydrofluorescein diacetate
DXM	dexamethasone
EP	E-prostanoid (prostaglandin E2 receptor)
ESI	electrospray ionization
FDR	false discovery rate
FBG	fasting blood glucose
GO	Gene Ontology
GSK-3β	glycogen synthase kinase-3 beta
HDL-C	high-density lipoprotein cholesterol
HO-1	heme oxygenase-1
ISO	isorhamnetin
KEGG	Kyoto Encyclopedia of Genes and Genomes
LC–MS	liquid chromatography–mass spectrometry
LDL-C	low-density lipoprotein cholesterol
Nrf2	nuclear factor erythroid 2–related factor 2
OPLS-DA	orthogonal partial least squares–discriminant analysis
PDB	Protein Data Bank
PI3K	phosphoinositide 3-kinase
PGE_2_	prostaglandin E_2_
PTGER2	prostaglandin E receptor 2 (EP2)
QC-RSD	quality-control relative standard deviation
ROS	reactive oxygen species
STZ	streptozotocin
T1DM	type 1 diabetes mellitus
UPLC	ultra-performance liquid chromatography
