# Supplementary material for: Isorhamnetin Exhibits Hypoglycemic Activity and Targets PI3K/AKT and COX-2 Pathways in Type 1 Diabetes
Source: Nutrients. 2025 Oct 11;17(20):3201. doi: 10.3390/nu17203201 (PMC12567359; doi:10.3390/nu17203201)
Supplement: Supplementary file 1 [file nutrients-17-03201-s001.zip › Figure_S2_Docking_interaction_details.pdf]

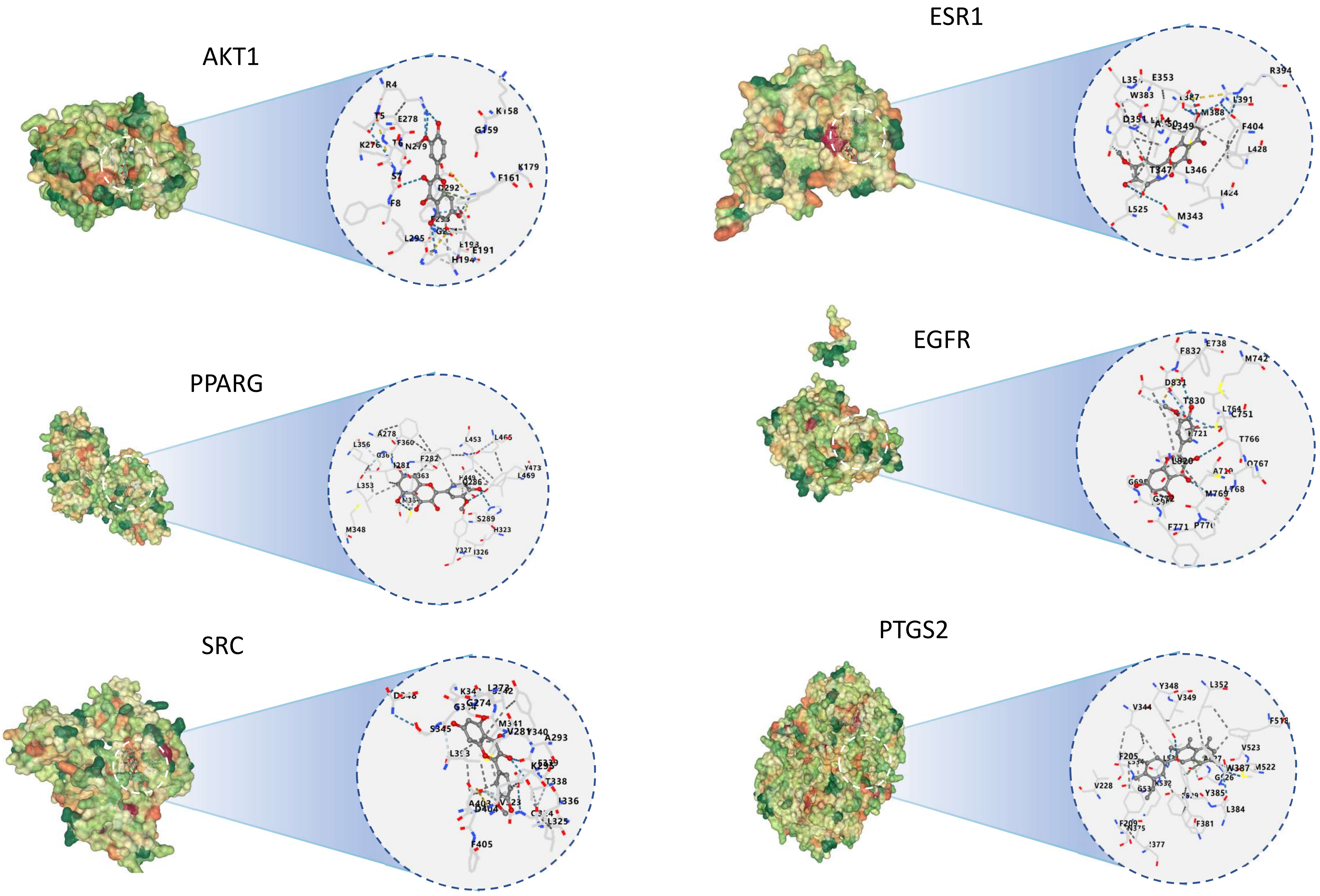
AKT1
ESR1
PPARG
EGFR
SRC
PTGS2

Type 类型	Color 颜色	Style 样式	Description 描述
Hydrogen Bond 氢键		- - -	Hydrogen-bond between strong donor and acceptor atoms.<br>强供体原子和受体原子之间的氢键。
Weak Hydrogen Bond 弱氢键		- - -	Hydrogen-bond between a carbon donor atom and an acceptor, or a Pi group and a donor atom.<br>碳供体原子和受体之间，或π基团和供体原子之间的氢键。
Hydrophobic Interaction 疏水相互作用		- - -	Interactions between alkyl groups, or a alkyl group and a Pi group.<br>烷基基团之间的相互作用，或烷基基团与π基团的相互作用。
Halogen Bond 卤键		- - -	Interactions with fluorine, chlorine, bromine or iodine atoms.<br>与氟、氯、溴或碘原子的相互作用。
Ionic Interaction 离子相互作用		- - -	Interactions between pairs of oppositely charged groups.<br>带相反电荷的基团对之间的相互作用。
Cation-Pi Interaction 阳离子-π相互作用		- - -	Interactions between a positively charged atom and the electrons of a delocalized Pi system.<br>一个带正电荷的原子与离域π体系的电子之间的相互作用。
Pi-Pi Stacking Pi-Pi 堆积		- - -	Interactions between delocalized Pi systems.<br>离域π体系之间的相互作用。

Type	Style	Description
Hydrogen Bond	- - -	Hydrogen-bond between strong donor and acceptor atoms.
Weak Hydrogen Bond	- - -	Hydrogen-bond between a carbon donor atom and an acceptor, or a Pi group and a donor atom.
Hydrophobic Interaction	- - -	Interactions between alkyl groups, or a alkyl group and a Pi group.
Halogen Bond	- - -	Interactions with fluorine, chlorine, bromine or iodine atoms.
Ionic Interaction	- - -	Interactions between pairs of oppositely charged groups.
Cation-Pi Interaction	- - -	Interactions between a positively charged atom and the electrons of a delocalized Pi system
Pi-Pi Stacking	- - -	Interactions between delocalized Pi systems.
