# Supplementary figures and images for: Isorhamnetin Exhibits Hypoglycemic Activity and Targets PI3K/AKT and COX-2 Pathways in Type 1 Diabetes
Source: Nutrients. 2025 Oct 11;17(20):3201. doi: 10.3390/nu17203201 (PMC12567359; doi:10.3390/nu17203201)

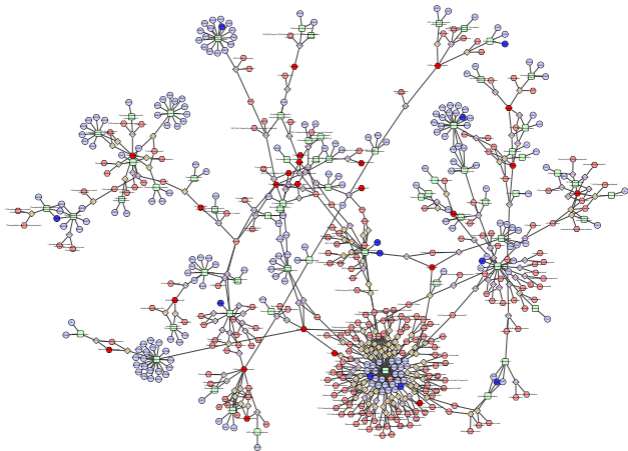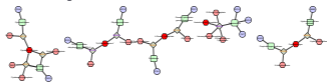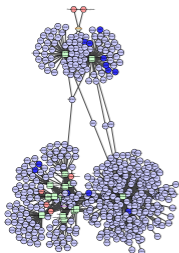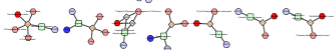

Supplement: Supplementary file 1 [file nutrients-17-03201-s001.zip › Figure_S1_Full_compound¿Cenzyme¿Cgene_network.pdf]
